# Volatile Compounds and Antioxidant and Antimicrobial Activities of Selected Citrus Essential Oils Originated from Nepal

**DOI:** 10.3390/molecules26216683

**Published:** 2021-11-04

**Authors:** Devi Prasad Bhandari, Darbin Kumar Poudel, Prabodh Satyal, Karan Khadayat, Sital Dhami, Dipa Aryal, Pratiksha Chaudhary, Aakash Ghimire, Niranjan Parajuli

**Affiliations:** 1 Biological Chemistry Lab, Central Department of Chemistry, Tribhuvan University, Kirtipur 44618, Nepal; dpbhandari_chem@yahoo.com (D.P.B.); darkwine51@gmail.com (D.K.P.); karankhadayat55@gmail.com (K.K.); Sitaldhami811@gmail.com (S.D.); dipaaryal260@gmail.com (D.A.); pratikshachaudhary274@gmail.com (P.C.); aakash.ghimire51@gmail.com (A.G.); 2 Department of Plant Resources, Natural Products Research Laboratory, Thapathali, Kathmandu 44600, Nepal; 3 Analytica Research Center, Kirtipur, Kathmandu 44600, Nepal; 4 Aromatic Plant Research Center, Lehi, UT 84043, USA

**Keywords:** limonene, antioxidant, enantiomeric distribution, antibacterial activity

## Abstract

*Citrus* species of plants are among the most commercially cultivated crops, mainly for their fruit. Besides, the generally consumed flesh inside the fruit, the peel is quite important too. Essential oils extracted from the peel have a history of being used by humankind for centuries. These essential oils are rich in antioxidants and antimicrobial agents. Comparative investigation of volatile constituents, and antioxidant and antimicrobial activities were undertaken. The essential oils were evaluated through gas chromatography-mass spectrometry (GC–MS), and enantiomeric composition by chiral GC–MS. Similarly, the antioxidant properties were evaluated by 2,2-diphenyl-1-picrylhydrazyl scavenging assay, and antimicrobial activities were assayed using the disk diffusion method. The highest extraction yield of 1.83% was observed in *Citrus sinensis* Osbeck. GC–MS analysis showed limonene (63.76–89.15%), γ-terpinene (0.24–6.43%), β-pinene (0.15–6.09%), linalool (0.35–3.5%), sabinene (0.77–2.17%), myrcene (0.74–1.75%), α-terpineol (0.28–1.15%), and α-pinene (0.2–0.58%) as the major constituents of the essential oil of the *Citrus* species studied. For the first time, through our study, chiral terpenoids have been observed from *Citrus grandis* Osbeck essential oil. The order of antioxidant activity is as follows: *Citrus grandis* Osbeck red flesh > *Citrus reticulata* Blanco > *Citrus sinensis* Osbeck > *Citrus grandis* Osbeck white flesh. Except for *Citrus grandis* Osbeck white flesh (52.34 µL/mL), all samples demonstrated stronger antioxidant activities than those of the positive control, quercetin (5.60 µL/mL). Therefore, these essential oils can be used as a safe natural antioxidant to prevent product oxidation. Likewise, citrus peel essential oil showed antimicrobial activity against tested bacterial strains, albeit marginal.

## 1. Introduction

*Citrus* species belong to the family Rutaceae and are among the most commercially significant crops cultivated in tropical and subtropical climate regions [1]. The peel of citrus fruit is a valuable raw material for the production of essential oils. Such oils have a history dating to very ancient times of being used in human society. At present, these oils find use in the perfume, food, and beverage industries. Likewise, there are cases of the use of such oils in folk and traditional medicines as well [2]. The extraction of these oils can be done through techniques such as hydrodistillation, cold pressing, and solvent extraction [3]. In addition, other techniques such as supercritical fluid extraction (SFE), pressurized liquid extraction (PLE), and microwave-assisted extraction (MAE) are also prevalent [4]. A high proportion (~93%) of citrus peel essential oil is commercially extracted by traditional methods such as steam distillation [5]. Citrus essential oils (CEOs) are particularly fascinating among essential oils since they can be used as antioxidants because of their ability to protect organisms and tissues from damage inflicted by reactive oxygen species, and also as flavoring agents [6,7]. These essential oils can be used as an alternative to synthetic preservatives since they are observed to display their antimicrobial and antioxidant activities with a broad spectrum of biological activities [8,9]. Further adding to their benefits, these essential oils are non-toxic, which is the reason explorations and studies on the antioxidant potential of essential oil are raising. However, it has been found that the emission of volatile components such as limonene, α/β-pinene, and camphene from citrus orchards contribute to ozone formation [10] in the troposphere and the deposition of ozone in the environment, contributing to the greenhouse effect [11].

CEOs are a complex mixture of volatile compounds belonging to terpenes and oxygenated terpenes [12]. Patterns of chemical composition in *Citrus* species differ with the origin, genotype, environmental factors, and method of extraction [13]. Moreover, volatile oil from *Citrus* species such as *C. reticulata*, *C. sinensis*, *C. paradisi*, *C. grandis*, *C. limon*, and *C. medica* mostly contain volatile chemicals such as limonene, α/β-pinene, sabinene, β-myrcene, limonene, linalool, α-humulene, and α-terpineol, which are responsible for antioxidant, anti-inflammatory, antifungal, antimicrobial, and wound-healing activities [1,14].

Multidrug-resistant pathogens are distributed globally and have directed the necessity of new antimicrobial agents, but the production has been delayed recently [15,16]. Essential oil, particularly from *Citrus* species, could be a possible candidate against such pathogens, particularly owing to their promising activity against pathogenic bacteria such as *Listeria* spp. [17], *Salmonella* spp. [18], Escherichia coli, *Staphylococcus aureus*, *Pseudomonas aeruginosa*, and *Bacillus subtilis* [19]. This is why CEOs can be linked to their high antimicrobial efficacy against various pathogens [20,21].

The analytical method used for the investigation of volatile chemistry in citruses is GC–MS, which helps in discrete complex mixtures of secondary metabolites produced by aromatic plants [14]. Along with GC–MS, an advanced technique of chiral GC–MS is used to obtain the chirality of the secondary metabolites. This is crucial for verifying the authenticity of the molecules and similarly aids in the identification of contaminants.

Despite the substantial data on peel oils of citrus from different geological locations of the world, there is a lack of information on the chemical composition and biological properties of Nepalese citrus. Therefore, this study sought to profile volatile compounds present in the epicarp of mandarin (*Citrus reticulata* Blanco), sweet orange (*Citrus sinensis* Osbeck), and pummelo (*Citrus grandis* Osbeck) from different geographical regions of Nepal, and access their antioxidant and antimicrobial activity alongside the enantiomeric distribution of chiral terpenoids, which may provide useful information regarding their authentication and associated impurities.

## 2. Results and Discussion

### 2.1. Isolation of Essential Oil and Yields

The extraction yield of essential oils from *Citrus* species varied from 0.5 to 1.83% in the study (Table 1), which depends on different factors such as regulation of biosynthetic genes, climatic variation, and expression of metabolites. Bourgou et al. reported 0.46 to 2.70% yield from *Citrus* species such as mandarin (2.7%) and orange (0.74%) [20]. Citrus peel grinding degree, hydrodistillation time, and added salts had an impact on *Citrus reticulata* Blanco peel reported from China [22]. Moreover, Tran et al. using the hydrodistillation to extract the essential oil from Vietnamese powdered mandarin (*Citrus reticulata* Blanco) at a temperature of 110–120 °C, reported the greatest yield of 5%, with a peel-to-solvent ratio of 1:4 (g/mL), and an extraction duration of 150 min indicating the size of the peel, the water-to-peel ratio, the temperature extraction, and the time extraction affecting extraction by hydrodistillation [23].

### 2.2. Chemical Composition of CEOs

**Figure 1:**
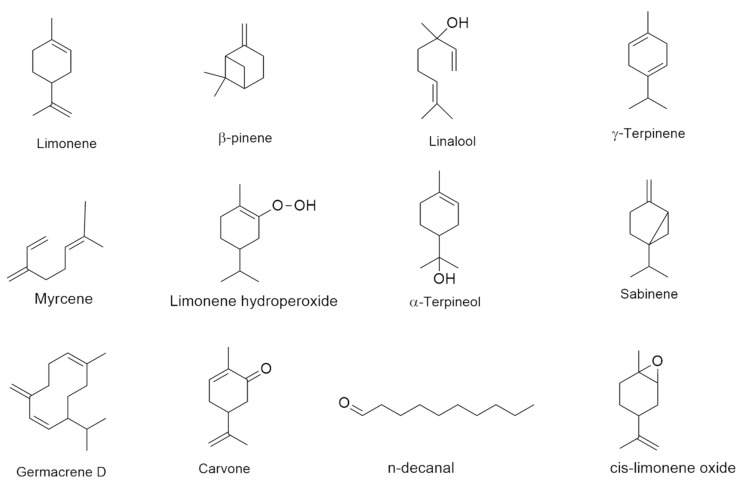
Major compounds in *Citrus* species.

The volatile constituents in CEOs were identified by GC–MS. The identified compounds are presented in Table 2 with their respective percentages. The result revealed that 50 components were present in sample *Citrus reticulata* Blanco (C1); among the major components were limonene (83.67%), γ-terpinene (6.09%), and linalool (2.65%). A previous study reported 53 components and the major compound was limonene. Thus, our finding is similar to a previous study on the essential oil of *Citrus reticulata* [22]. A total of 47 components were identified in *Citrus sinensis* Osbeck (C2) accounting for the total percentage of essential oil. Limonene (86.59%) and linalool (3.50%) were the main components. Previous studies showed that limonene was the main constituent of *Citrus sinensis* Osbeck essential oil [24]. Analysis by GC–MS identified, in the essential oil of *Citrus grandis* Osbeck red flesh (C3), 55 components, and limonene (63.76%), β-pinene (6.09%), and limonene hydroperoxide 1 (2.66%) were the main components. Analysis of *Citrus grandis* Osbeck white flesh (C4) essential oil relieved 53 components where limonene (89.15%) and β-pinene (2.51%) were the most abundant. This is comparable to the results of earlier research on *Citrus grandis* Osbeck, in which a total of 33 compounds, with the major component limonene (87.5%), were identified [25]. From a previous report, a similar range of limonene was observed [26]. Extraction methods used can influence the number of volatile components present in the CEOs [27]. In this study, most secondary metabolites belong to the monoterpene class followed by oxygenated monoterpene. Major compounds in the *Citrus* species are shown in Figure 1.

### 2.3. Enantiomeric Composition of CEOs

In total, 8, 6, 8, and 7 chiral compounds were identified in *Citrus reticulata* Blanco, *Citrus sinensis* Osbeck, *Citrus grandis* Osbeck red flesh, and *Citrus grandis* Osbeck white flesh (C1, C2, C3, and C4), respectively. Relative percentages of the levorotatory and dextrorotatory compounds in the essential oil from different species of citrus are listed in Table 3. Limonene is a major monoterpene that has (+)-limonene in our study. Limonene, linalool, and germacrene D exist in dextrorotatory form. On the other hand, the levorotatory enantiomer predominated for β-pinene, carvone, and *trans*-carveol while the enantiomeric distribution varied for α-pinene, sabinene, and α-terpineol depending upon the *Citrus* species. This study reports for the first time chiral terpenoids from *Citrus grandis* Osbeck (C3 and C4) essential oil.

### 2.4. Antioxidant Activity of CEOs

Literature shows that extracts from the peel of citrus had the highest antioxidant activity compared to essential oil. In the present study, DPPH radical scavenging assay was used for the determination of the antioxidant activity of citrus peel essential oil by comparing it with the activity of quercetin. The secondary metabolites of citrus peel essential oil can donate electrons; it changes the purple color of DPPH to yellow (diphenyl-picrylhydrazine) for quantification of antioxidant activity. The IC_50_ value is a commonly used criterion for determining the antioxidant activity of test samples. It is estimated as the antioxidant concentration is needed to minimize the initial DPPH concentration by 50% [28]. The order of antioxidant activity follows as *Citrus grandis* Osbeck red flesh, *Citrus reticulata* Blanco, *Citrus sinensis* Osbeck, and *Citrus grandis* Osbeck white flesh (C3 > C1 > C2 > C4), depicting *Citrus grandis* Osbeck red flesh as the highest antioxidant activity. The lowest IC_50_ value represents the highest scavenging activity of the essential oil as shown in Table 4. However, the *Citrus grandis* Osbeck white flesh (C4) sample demonstrated weaker antioxidant activities than that of positive control quercetin (5.60 ± 0.42 µL/mL).

The major component of essential oil, limonene and α-pinene, has shown bioactive properties such as antioxidant, antimicrobial, and antiulcer activities [29]. Limonene, due to the donation of hydrogen atoms, was converted into a stable form from free radical and the potential of antioxidant effect was determined which was directly proportional to the antioxidant activity that was reported previously [30]. However, in our study, *Citrus grandis* Osbeck white flesh (C4) has a greater percentage of limonene and monoterpene but has weaker antioxidant properties, which can be attributed that the overall performance as an antioxidant, which is the result of compounded interaction among components and oxidizable substance to be protected. Depending upon the experimental condition and essential oil composition, synergistic or antagonistic behavior is expected and exceptions may be obtained [31] and this could be an area of further exploration in CEO. Moreover, phenolic compounds such as polyphenols and phenylpropanoids, which are sometimes among the main components of essential oil, have been found to have higher antioxidant values [31,32]. Additionally, the major constituents of CEO, limonene, and β-pinene, have a grounded defensive activity against many kinds of malignant growth as reported in the literature [33].

### 2.5. Antibacterial Activity

CEOs have demonstrated a broad range of antibacterial activity against different classes of pathogens [34,35,36]. The antibacterial activity of the CEOs was tested against *Staphylococcus aureus*, *Escherichia coli*, *Klebsiella pneumoniae*, *Shigella sonnei*, and *Salmonella typhi* in this study. The effectiveness of antibacterial activities of various CEOs was evaluated by measuring the zone of inhibition (ZoI), summarized in Table 5. The result indicates that the CEO showed a moderate to poor antibacterial activity at 25% strength against test bacterial strain. Our study illustrated that *Citrus grandis* Osbeck white flesh (C4) has higher activity against *Staphylococcus aureus* and *Escherichia coli* whereas the *Citrus reticulata* Blanco (C1) sample showed noticeable activity against *Salmonella typhi*. The tested CEOs demonstrated weaker antibacterial activity as compared to neomycin. A previous study done on CEOs showed a higher ZoI against Gram-positive and Gram-negative bacteria than in our study, which might be due to the harvest of the sample at different ripening stages that significantly affects the antibacterial activity [20,21]. Additionally, limonene significantly inhibits antibacterial activity and can rupture the cell membrane of bacteria and lead to a change in morphology [37].

## 3. Materials and Methods

### 3.1. Plant Material

**Figure 2:**
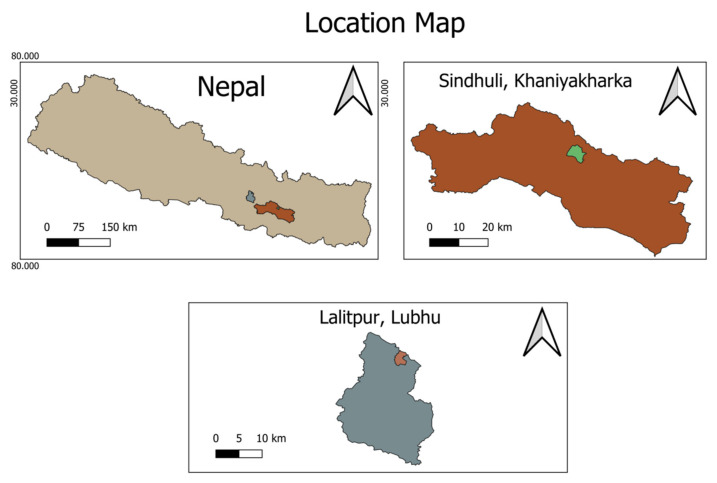
The geographical location of *Citrus* species collection site.

The fruit peel of *Citrus* species was collected in the area presented in Figure 2. It was collected at the beginning of the ripening period (November 2020), and fruit was identified by a horticulturist at the National Center for Fruit Development, Kirtipur, Kathmandu. Voucher specimens of the plant have been deposited at National Herbarium and Plant Laboratories (KATH), Government of Nepal, Lalitpur, Nepal.

### 3.2. Isolation of Essential Oil

Fresh fruit peel of citrus was cut into small pieces and essential oil was extracted by hydrodistillation in Clevenger apparatus as previously described [38]. The essential oil was dried using anhydrous sodium sulfate and was stored in bottles at 4 °C until use in further studies. The percentage yield of essential oil of different samples is summarized in Table 1.

### 3.3. Chemical Composition Analysis by GC–MS

Analysis of the volatile chemical constituents in the CEOs was carried out using a GC–MS-QP2010 Ultra gas chromatograph-mass spectrometer (Shimadzu, Columbia, MD, USA) under the following conditions: mass selective detector (MSD), operated in the EI mode (electron energy = 70 eV), with scan range = 40–400 *m*/*z*, and scan rate = 3.99 scans/s. The GC column was a ZB-5 ms fused silica capillary with a (5% phenyl)-polydimethylsiloxane stationary phase, a film thickness of 0.25 μm, a length of 60 m, and an internal diameter of 0.25 mm. The carrier gas was helium (80 psi) with a column head pressure of 48.7 kPa and a flow rate of 1.0 mL/min. The injector temperature was 260 °C, and the detector temperature was 280 °C. The GC oven temperature was programmed as follows: hold a 40 °C initial temperature for 10 min; followed by an increase in temperature at 3 °C/min to 200 °C, and then increased at 2 °C/min to 260 °C. A 5% *w*/*v* solution of each CEO sample in dichloromethane was prepared, and 1 μL of the sample was injected using a 30:1 split ratio [38,39]. Identification of compounds was based on the retention indices determined by reference to a homologous series of n-alkanes and by comparison of the mass spectral fragmentation patterns. Relative percentages of the individual components of CEOs and different classes of compounds (%) are listed in Table 2.

### 3.4. Enantiomeric Analysis by Chiral GC–MS

Shimadzu GC–MS-QP2010S with EI mode (70 eV) and B-Dex 325 chiral capillary GC column was used to perform enantiomeric analysis of CEO samples. It scans in the 40–400 *m*/*z* range at a scan rate of 3.0 Scan/s. The column temperature was set at 50 °C, initially increased by a rate of 1.5 °C/min till 120 °C and then at 2 °C/min until 200 °C. The final temperature of the column was 200 °C and was kept constant. The carrier gas was helium with a constant flow rate of 1.8 mL/min. For each essential oil sample, 3% *w*/*v* solution in DCM was prepared, and 0.1 μL was injected using a split ratio of 1:45 [38,40,41]. The enantiomer percentages were determined from the peak area. Comparison of retention times and mass spectral fragmentation patterns with authentic samples obtained from Sigma-Aldrich (Milwaukee, WI, USA) was used to identify the enantiomers. Table 3 shows the enantiomeric distribution of components in the CEOs.

### 3.5. Antioxidant Activity of CEOs

The antioxidant activity of the CEOs was measured using the DPPH radical scavenging assay which was modified from the colorimetric method [42,43]. Briefly, 0.1 mM of DPPH solution was prepared in methanol and 100 µL of this solution was mixed with 100 µL of essential oil at different concentrations. The solution was then incubated at room temperature for 30 min. in dark and the absorbance was measured at 517 nm using a microplate reader (Epoch2, BioTek, Instruments, Inc., Winooski, VT, USA). Quercetin was used as a positive control and 30% DMSO was used as a negative control. The inhibitory concentration (IC_50_) was calculated from the plot of inhibition percentage, against sample concentration. Tests were carried out in triplicate. The calculated IC_50_ values are showcased in Table 4.

### 3.6. Antibacterial Activity of CEOs

The antimicrobial activity of the CEOs was done by the disc diffusion method as previously described by Puškárová et al. [44]. The bacterial inoculum was prepared in a Mueller Hinton broth by adding a single colony of bacteria from an overnight culture plate. Then it was incubated at 37 °C until the turbidity matched with 0.5 McFarland. The lawn culture was done in a pre-warmed sterile Mueller Hinton agar plate placed at the same temperature using sterile cotton swabs. The sterile filter paper discs (6 mm) prepared from Whatman No. 1 were pressed gently onto the surface of the agar plates and essential oil at 25% strength (diluted in DMSO) was pipetted onto the discs. The plates were then incubated at 37 °C for 24 h and the ZoI along with disc was measured in mm. Neomycin was used as positive control and DMSO was used as a negative control. The volume that comprised 10 mg pure essential oil was used to test antibacterial activity as each essential oil varied in density. The antibacterial activity of the CEOs is displayed in Table 5.

### 3.7. Data Analysis

The results were processed using BioTek Gen5 Microplate Data Collection & Analysis Software (BioTek, Instruments, Inc., Winooski, VT, USA) which was subsequently analyzed using Microsoft Excel. GraphPad Prism version 8 was used to calculate the IC_50_ value of essential oil. The results are presented as the mean ± standard deviation of the mean of triplicate data.

## 4. Conclusions

The essential oils from *Citrus* species of Nepalese origin have shown variation in chemical compositions, enantiomeric distributions, and antioxidant and antimicrobial activities. Limonene (63.76–89.15%) is a major component in all essential oils; it exists in dextrorotatory form, and its abundance is found to be in the order *Citrus grandis* Osbeck white flesh, *Citrus grandis* Osbeck red flesh, *Citrus sinensis* Osbeck, and *Citrus reticulata* Blanco (C4 > C3 > C2 > C1). Through this study, chiral terpenoids have been for the first time reported being found in essential oils produced from the peels of *Citrus grandis* Osbeck (C3 and C4). Furthermore, it is of interest that the peel of *Citrus sinensis* Osbeck has shown a higher extraction yield. The yield of extraction, its constituents, and ultimately the biological properties depends upon factors such as genetic variation and extraction procedure. The obtained results of the chiral and GC–MS analysis of these samples can be used as a fingerprint to detect adulteration and authentication issues. Furthermore, out of four CEOs: *Citrus reticulata* Blanco (C1), *Citrus sinensis* Osbeck (C2), and *Citrus grandis* Osbeck red flesh (C3) demonstrated strong antioxidant capacity, suggesting that they may be used as a natural antioxidant to prevent product oxidation. Despite showing promising antioxidant activities, the same show only marginal antimicrobial properties. Through this study, we have laid a path that leads to the commercial production of CEOs in Nepal, because of the observation of similar volatile chemotype and biological properties to other origins.

## Figures and Tables

**Table 1:** Extraction yield of essential oil in citrus peel.

Plant	Extraction Method	GPS Coordinates	Extraction Yield (*v*/*w*%)
Mandarin *Citrus reticulata* Blanco (C1)	Hydrodistillation	27°16′59.0″ N, 85°58′46.5″ E	0.5
Sweet Orange *Citrus sinensis* Osbeck (C2)	Hydrodistillation	27°16′59.0″ N, 85°58′46.5″ E	1.83
Pummelo *Citrus grandis* Osbeck red flesh (C3)	Hydrodistillation	27°38′14.5″ N, 85°22′04.7″ E	1.0
Pummelo *Citrus grandis* Osbeck white flesh (C4)	Hydrodistillation	27°38′14.2″ N, 85°22′04.6″ E	1.5

Note: *Citrus grandis* Osbeck (red flesh) and *Citrus grandis* Osbeck (white flesh) are cultivars of *Citrus grandis* Osbeck.

**Table 2:** Essential oil compositions of citrus essential oil.

Retention Index	Compounds	Mandarin *Citrus reticulata* Blanco (C1) %	Sweet Orange *Citrus sinensis* Osbeck (C2) %	Pummelo *Citrus grandis* Osbeck Red Flesh (C3) %	Pummelo *Citrus grandis* Osbeck White Flesh (C4) %
924	α-Thujene	0.1	0.04	0.02	0.04
931	α-Pinene	0.57	0.2	0.58	0.42
948	Camphene	0.04	0.03	0.06	0.05
972	Sabinene	1.3	0.77	2.17	2.17
978	β-Pinene	0.58	0.15	6.09	2.51
983	6-Methyl-5-hepten-2-one	-	-	0.15	-
989	Myrcene	1.59	1.18	0.74	1.75
1002	*n*-Octanal	0.19	0.28	0.06	-
1003	Para-mentha-1(7),8-diene	-	-	-	0.01
1008	α-Phellandrene	0.05	0.04	-	0.03
1008	δ-3-Carane	0.03	0.04	-	0.03
1016	α-Terpinene	0.13	0.04	-	0.11
1024	*p*-Cymene	0.24	0.03	0.32	0.03
1028	Limonene	83.67	86.59	63.76	89.15
1029	β-phellandrene	-	0.35	-	-
1031	1,8-Cineole	-	0.07	0.54	-
1034	*Cis*-β-ocimene	0.04	-	-	0.04
1044	*Trans*-β-ocimene	0.24	0.13	-	0.43
1058	γ-Terpinene	6.43	0.64	-	0.24
1062	3-Methyl decane	-	-	-	0.01
1067	*Cis*-linalool oxide	-	-	0.53	0.08
1069	*n*-Octanol	0.1	0.19	-	-
1073	Pinol	-	-	0.02	-
1086	Terpinolene	0.34	0.08	-	0.06
1086	*Trans*-linalool oxide	-	0.04	0.37	0.04
1091	*p*-Cymenene	0.01	-	-	0.01
1098	Perillene	-	-	0.07	-
1099	Linalool	2.65	3.5	1.87	0.35
1105	*n*-Nonanal	0.08	0.14	-	-
1112	2-Methyl-6-methylen-octa-1,7-dien-3-one	0.02	0.05	-	-
1122	*Trans*-*p*-Mentha-2,8-dien-1-ol	0.01	-	0.34	0.02
1124	Cyclooctanone	-	-	0.07	-
1124	*Cis*-para-menth-2-en-1-ol	-	-	-	0.01
1130	Limona ketone	-	-	0.05	-
1133	*Cis*-limonene oxide	0.01	-	2.02	-
1137	*Trans*-limonene oxide	0.02	-	1.31	-
1137	*Cis*-para-mentha-2,8-dien-1-ol	0.01	-	-	0.02
1138	Geijerene	0.03	-	-	-
1140	*Trans*-pinocarveol	-	-	0.11	-
1145	Camphor	0.01	-	0.08	-
1152	Citronellal	0.1	0.05	-	-
1163	Pinocarvone	-	-	0.22	-
1166	6-Methyl-bicyclo [3.3.0] oct-2-en-7-one	-	-	0.12	-
1175	Menthol	-	0.05	-	-
1178	Naphthalene	0.01	-	-	0.01
1179	Para-1,8-menthadien-4-ol	0.01	-	0.12	-
1180	Terpinen-4-ol	0.2	0.51	0.33	0.13
1184	Para-Methyl acetophenone	-	-	0.02	-
1186	Cryptone	-	-	0.17	-
1191	Methyl salicylate	0.01	0.14	0.76	-
1195	α-Terpineol	0.29	0.92	1.15	0.28
1197	γ-Terpineol	-	-	0.29	-
1197	*Cis*-Piperitol	-	-	0.1	0.03
1207	*n*-Decanal	0.4	1.14	-	-
1209	Octyl acetate	-	0.09	-	-
1218	*Trans*-carveol	0.01	0.07	0.86	0.01
1223	Nerol	0.02	0.03	-	0.01
1226	Citronellol	0.06	-	-	-
1229	Thymol methyl ether	0.11	-	-	-
1232	*Cis*-Carveol	-	-	-	0.03
1238	Neral	0.01	0.18	0.08	-
1242	Carvone	0.01	0.02	1.3	-
1250	Geraniol	-	-	-	0.04
1253	Piperitone	-	0.06	0.31	-
1258	Methyl-dodecane	-	-	-	0.01
1266	*n*-Decanol	0.01	-	-	-
1268	Geranial	-	0.32	0.13	-
1270	Isopiperitenone	-		0.14	-
1278	Perilla aldehyde	0.04	0.1	0.15	-
1282	Bornyl acetate	-	-	0.04	0.02
1285	Limonene dioxide	-	-	0.23	-
1287	Mentha-dienehydroperoxide	-	-	2.53	-
1289	Thymol	0.08	-	-	-
1309	4-Vinyl guaiacol	-	0.08	-	-
1317	Limonene hydroperoxide	-	-	2.39	-
1329	Para-mentha-1,8-dien-4-hydroperoxide	-	-	1.1	-
1331	Bicycloelemene	-	-	-	0.02
1334	δ-Elemene	0.04	0.04	-	0.07
1348	*Iso*Neryl-acetate	-	-	-	0.01
1353	Terpena-diol	-	-	0.05	-
1354	Limonene hydroperoxide 1	-	-	2.66	-
1357	Eugenol	-	-	-	0.01
1357	*Cis*-Carvyl acetate	-	-	-	0.01
1359	Neryl acetate	-	-	-	0.01
1373	Limonene hydroperoxide 2	-	-	2.04	-
1376	α-Copaene	-	0.04	-	0.01
1377	Geranyl acetate	-	0.03	-	0.3
1391	β-Elemene	0.01	-	-	0.03
1405	*Cis*-caryophyllene	-	-	0.09	-
1409	Dodecanal	0.03	0.17	-	-
1418	β-Caryophyllene	-	0.07	0.16	0.06
1430	γ-Elemene	0.02	-	-	-
1431	β-Copaene	-	0.04	0.07	0.04
1433	Perillyl acetate	-	-	-	0.04
1484	Germacrene D	0.03	0.05	-	1.05
1496	Valencene	-	0.06	-	-
1496	Bicyclogermacrene	-	-	-	0.08
1500	α-Muurolene	-	-	-	0.01
1504	*Trans*-*trans*-α-farnesene	-	1.97	-	-
1521	δ-cadinene	-	0.06	-	0.01
1560	Germacrene B	0.03	-	-	0.01
1576	Spathulenol	-	-	0.08	-
1578	Caryophyllene oxide	-	-	0.12	-
	Monoterpenes	95.37	90.3	74.69	97.08
	Oxygenated monoterpenes	3.64	5.17	23.12	1.44
	Sesquiterpene	0.13	2.33	0.32	1.39
	Oxygenated sesquiterpenes	0	0	0.25	0.03
	Others	0.86	2.2	1.62	0.06

**Table 3:** Enantiomeric distributions of chiral terpenoid of CEOs.

Chiral Terpenoids	Citrus Peel Essential Oil along with Enantiomeric Composition							
	Mandarin *Citrus reticulata* Blanco (C1)		Sweet Orange *Citrus sinensis* Osbeck (C2)		Pummelo *Citrus grandis* Osbeck (Red Flesh) (C3)		Pummelo *Citrus grandis* Osbeck (White Flesh) (C4)	
	−	+	−	+	−	+	−	+
α-Pinene	29.08	70.92	0	100	53.89	46.11	27.13	72.87
Sabinene	7.32	92.68	0	100	40.5	59.5	18.04	81.96
β-Pinene	34.33	65.67	-	-	99.15	0.85	97.14	2.86
Limonene	0.9	99.1	0.59	99.41	0.87	99.13	0.69	99.31
Linalool	3.19	96.81	11.19	88.81	31.69	68.31	38.94	61.06
α-Terpineol	46.11	53.89	10.12	89.88	58.64	41.36	42.61	57.39
Germacrene D	-	-	-	-	-	-	5.86	94.14
Carvone	-	-	-	-	50.86	49.14	-	-
*trans*-Carveol	-	-	-	-	59.2	40.8	-	-
Terpinen-4-ol	53.97	46.03	28.69	71.31	-	-	-	-
α-Thujene	76.86	23.17	-	-	-	-	-	-

**Table 4:** Antioxidant activity of Citrus essential oil.

Citrus Essential Oil	IC_50_ (µL/mL)
Mandarin *Citrus reticulata* Blanco (C1)	2.30 ± 0.37
Sweet Orange *Citrus sinensis* Osbeck (C2)	3.33 ± 0.66
Pummelo *Citrus grandis* Osbeck red flesh (C3)	1.56 ± 0.08
Pummelo *Citrus grandis* Osbeck white flesh (C4)	52.34 ± 0.62
Quercetin (Positive control)	5.60 ± 0.42

**Table 5:** Antibacterial activity of citrus essential oil.

Essential Oil	Bacterial Strains (ZoI) in mm				
	*S. aureus* ATCC 25923	*E. coli* ATCC 25922	*K. pneumoniae* ATCC 700603	*S. sonnei* ATCC 25931	*S. typhi* ATCC 14028
Mandarin *Citrus reticulata* Blanco (C1)	9	9	9	9	10
Sweet Orange *Citrus sinensis* Osbeck(C2)	8	7	10	8	9
Pummelo *Citrus grandis* Osbeck (red flesh) (C3)	8	8	8	10	9
Pummelo *Citrus grandis* Osbeck (white flesh) (C4)	11	11	9	10	8
Positive control	23	19	16	30	19

Note: Positive control: Neomycin 30 µg/disc and Negative control: DMSO.

## Data Availability

All data are available in the manuscript.
